# Elevated Cell-Specific Microparticles Are a Biological Marker for Cerebral Dysfunctions in Human Severe Malaria

**DOI:** 10.1371/journal.pone.0013415

**Published:** 2010-10-14

**Authors:** Joël Bertrand Pankoui Mfonkeu, Inocent Gouado, Honoré Fotso Kuaté, Odile Zambou, Paul Henri Amvam Zollo, Georges Emile Raymond Grau, Valéry Combes

**Affiliations:** 1 Department of Biochemistry, Faculty of Science, University of Douala, Douala, Cameroon; 2 Laboratory service, Laquintinie Hospital, Douala, Cameroon; 3 Pediatric service, Deido district Hospital, Douala, Cameroon; 4 Department of Pathology, The University of Sydney, Sydney, New South Wales, Australia; Université Pierre et Marie Curie, France

## Abstract

Cerebral malaria (CM) and severe anemia (SA) are the most severe complications of *Plasmodium falciparum* infections. Although increased release of endothelial microparticles (MP) correlates with malaria severity, the full extent of vascular cell vesiculation remains unknown. Here, we characterize the pattern of cell-specific MP in patients with severe malaria. We tested the hypothesis that systemic vascular activation contributes to CM by examining origins and levels of plasma MP in relation to clinical syndromes, disease severity and outcome. Patients recruited in Douala, Cameroon, were assigned to clinical groups following WHO criteria. MP quantitation and phenotyping were carried out using cell-specific markers by flow cytometry using antibodies recognizing cell-specific surface markers. Platelet, erythrocytic, endothelial and leukocytic MP levels were elevated in patients with cerebral dysfunctions and returned to normal by discharge. In CM patients, platelet MP were the most abundant and their levels significantly correlated with coma depth and thrombocytopenia. This study shows for the first time a widespread enhancement of vesiculation in the vascular compartment appears to be a feature of CM but not of SA. Our data underpin the role of MP as a biomarker of neurological involvement in severe malaria. Therefore, intervention to block MP production in severe malaria may provide a new therapeutic pathway.

## Introduction

Malaria still poses a serious threat to human life. According to the WHO 2008 world malaria report, of 3.3 billion people at risk in 2006, there were an estimated 247 million malaria cases causing nearly a million deaths, mostly of children under 5 years. 109 countries were endemic for malaria in 2008, 45 within the WHO African region (WHO report http://apps.who.int/malaria/wmr2008/) [1]. Severe malarial anemia (SA) and cerebral malaria (CM) are the most severe complications of infections with *Plasmodium falciparum* (Pf). Insights into the processes leading to these severe forms might lead to new interventions that address pathophysiological processes causing malaria's peculiar morbidity and mortality [2],[1].

One of the hypotheses to explain the severe malaria excessive response is the “all immunological theory”. It rests on the accumulation of host cells (including parasitized erythrocytes) in the brain microvasculature of CM patients. These cells, especially mononuclear leucocytes and platelets, induce an increased cytokine and chemokine production. In turn, these inflammatory processes result in an increased cell activation, which may be reflected, among other changes, by elevated circulating microparticle levels [3].

Microparticles (MP), also referred to as microvesicles, are fragments physiologically shed from the plasma membrane of virtually all cell types. MP are also released during *in vitro* cell stimulation and are a marker of cellular activation and apoptosis or tissue damage occurring *in vivo* in a variety of pathophysiological circumstances [4],[5].

Investigations on vesiculation during malaria infection in murine models have generated valuable data about the physiopathology of severe malaria [6],[7]. As none of the animal or *in vitro* models exactly mimic the human disease; it is essential to investigate these processes in malaria patients for better understanding [8],[9],[10]. In this line of thought, platelet accumulation has been identified in the brain microvasculature of patients with CM, suggesting that as in experimental models, they could be pathogenic in the neurological syndrome [11]. Recently, a dramatic increase in plasma levels of MP of endothelial origin has been found in falciparum-infected Malawian children, specifically in patients with CM, as opposed to patients with severe malarial anemia (SA) or uncomplicated malaria (UM) [12]. As cell types other than endothelial cells, including platelets, monocytes, T lymphocytes and red blood cells can release MP, it is essential to explore the diverse cellular origins of MP and to evaluate the extent to which vesiculation correlates with clinical and biological parameters.

The objective of the present work was thus twofold: firstly, to characterize and compare MP and their phenotypes in patients with severe malaria as opposed to those with UM or healthy controls and, secondly, to examine MP phenotypes in relation to clinical syndromes, disease severity and outcome.

## Materials and Methods

### Recruitment of patients

The patient recruitment throughout the year 2007 consisted of the enrolment of children 0 to 15 years old who presented to participating health institution for health problems in Douala (Cameroon). Children with diarrhea, non malaria infections and HIV were excluded. Finally, children who met the study inclusion criteria were recruited after informed consent and, at a later time allocated to the different malaria severity groups.

Table 1 provides anthropometric, clinical and hematological characteristics of the subjects in the 5 groups (UM, SA, CM, CM+SA and controls). These figures and clinical management have been presented in more detail elsewhere [13]. Briefly, children with UM, SA and CM were given quinine base for 3 days. SA patients also received whole blood prior to quinine and iron supplementation. CM patients received, after quinine, an artemisinin combination therapy and iron supplementation. The study protocol was approved by the Cameroon Bioethics committee and the Provincial delegation of Public Health. Written consent (informed consent form) was obtained from most parents/guardians of study participants. In some cases, only verbal consent was obtained due to illiteracy in the presence of the physician in charge. Such process is acceptable by the Cameroon Bioethic committee, as it does not consider the written consent superior in anyway to the verbally given consent in circumstances like illiteracy.

**Table 1:** Anthropometric, clinical and hematological characteristics of subjects in the different groups.

Groups	Controls	UCM	SMA	CM	CM&SMA
n	30	59	45	32	10
Male	9	34	27	15	7
Female	21	25	18	17	3
Age (months)	59.07±8.82*	41.56±5.12	49.93±5.71	26.13±2.41	36.7±8.98
Weight (Kg)	19.05±1.47	15.68±1.22	15.63±1.09	12.18±0.65	13.25±1.27
Temperature (°C)	37.19±0.04	38.91±0.09	38.63±0.10	39.83±0.16	39.57±0.33
FRT (hours)	N/A	34.40±2.68	39.17±4.08	58.11±5.58	96±6.41
BCS	5±0	4.75±0.06	4.42±0.10	1.66±0.10	1.70±0.15
CRT (hours)	N/A	N/A	N/A	14.10±1.40	27±3.71
PRT(hours)	N/A	19.05±2.80	46.06±4.24	52.67±4.67	90±12.63
Fits frequencies	N/A	0.89±0.23	1.28±0.47	1.62±0.56	1.44±0.86
Parasites (/µl)	N/A	14518±2443	67345±13512	138007±28183	141000±50784
WBC (/µl)	7360±199	9497±330	12152±537.6	13786±638	14610±1174
RBC (X 10^3^/µl)	4179±104	3986±55.58	2292±88.40	3753±101	2626±190
Hgb (g/dl)	12.52±0.15	10.97±0.16	5.04±0.21	10.56±0.19	6.42±0.42
Platelets (/µl)	204780±10199	207788±7529	190149±11671	123469±6796	109000±27388

n: number;

*: Mean±SE; FRT: Fever Resolution Time; BCS: Blantyre Coma Score; CRT: Coma Resolution Time; PRT: Prostration Resolution Time; WBC: White blood cells count; RBC: Red blood cells count; Hgb: Hemoglobin; N/A: Not applicable.

### Definition of categories

SA was defined as a hemoglobin<8.0 g/dl or a hematocrit<18 in a patient with a positive malaria smear. CM was diagnosed if a patient with a positive smear presented with impaired consciousness as measured by a Blantyre coma score [14] ≤2 (range: 0–5) and had a normal cerebrospinal fluid. Children without any of the above mentioned symptoms, but presenting with usual malaria symptoms and a positive smear were classified as UM.

### Malaria diagnosis

Positive Pf malaria diagnosis was performed as described elsewhere [13]. Briefly, diagnosis was made on a thick blood film. For control subjects, in addition to the thick film, a more sensitive antigenic test to detect PfHRP2 (*P. falciparum*-specific Histidin Rich Protein 2) was performed using ParaHit dipstick (Span Diagnostics Ltd, India).

### Sample collection and processing for MP analysis

Two milliliters of blood were collected by cubital venipuncture in 0.124 M trisodium citrate tube. Within 3 h of collection, platelet free plasma was prepared as previously described [7] then stored at −80°C. At discharge, another blood collection was performed in children with severe malaria (CM and SA groups). These aliquots were shipped on dry ice to the Vascular Immunology Unit, Sydney.

### Microparticle analysis

#### mAbs and reagents

FITC-annexin V, PE-mAbs/isotype-matched controls (CD3, CD11b/Mac1, CD41/GPIIbIIIa, CD105/endoglin, CD235a/glycophorin A), as well as FITC-antibodies/isotype-matched controls (CD51/α_v_ subunit of α_v_β3 complex) were all from Beckman-Coulter-Immunotech, Marseille, France. Annexin V binding buffer (BB) (10×) and Flowcount® fluorospheres were from Beckman-Coulter, Fullerton, CA.

#### MP labeling

Total MP (T-MP:annexin V^+^) numbers were determined by detection of phosphatidylserine (PS) using FITC-annexin V diluted 1∶1 in 10× BB. In parallel, the cellular origin of these MP was investigated using cell-specific mAbs. Leukocyte MP were detected using anti-CD3 (lymphocyte, L-MP:CD3^+^) and anti-CD11b (monocyte, M-MP:CD11b^+^); platelet MP with anti-CD41 (P-MP:CD41^+^), red blood cell MP with anti-CD235a (R-MP:CD235a^+^) and endothelial MP with PE-anti-CD105 and the FITC-anti-CD51 (E-MP:CD105^+^ and E-MP:CD51^+^, respectively). The latter was used in order to further characterize and confirm our previous study on endothelial MP in Malawi [12].

Briefly, platelet free plasma was diluted in PBS (1∶9, V/V), labeled and resuspended in 200 µl of PBS or BB for annexin V (1×). 20 µl of Flowcount® beads (1000/µl) were added to each well as an internal standard.

#### Flow cytometry analysis

**Figure 1:**
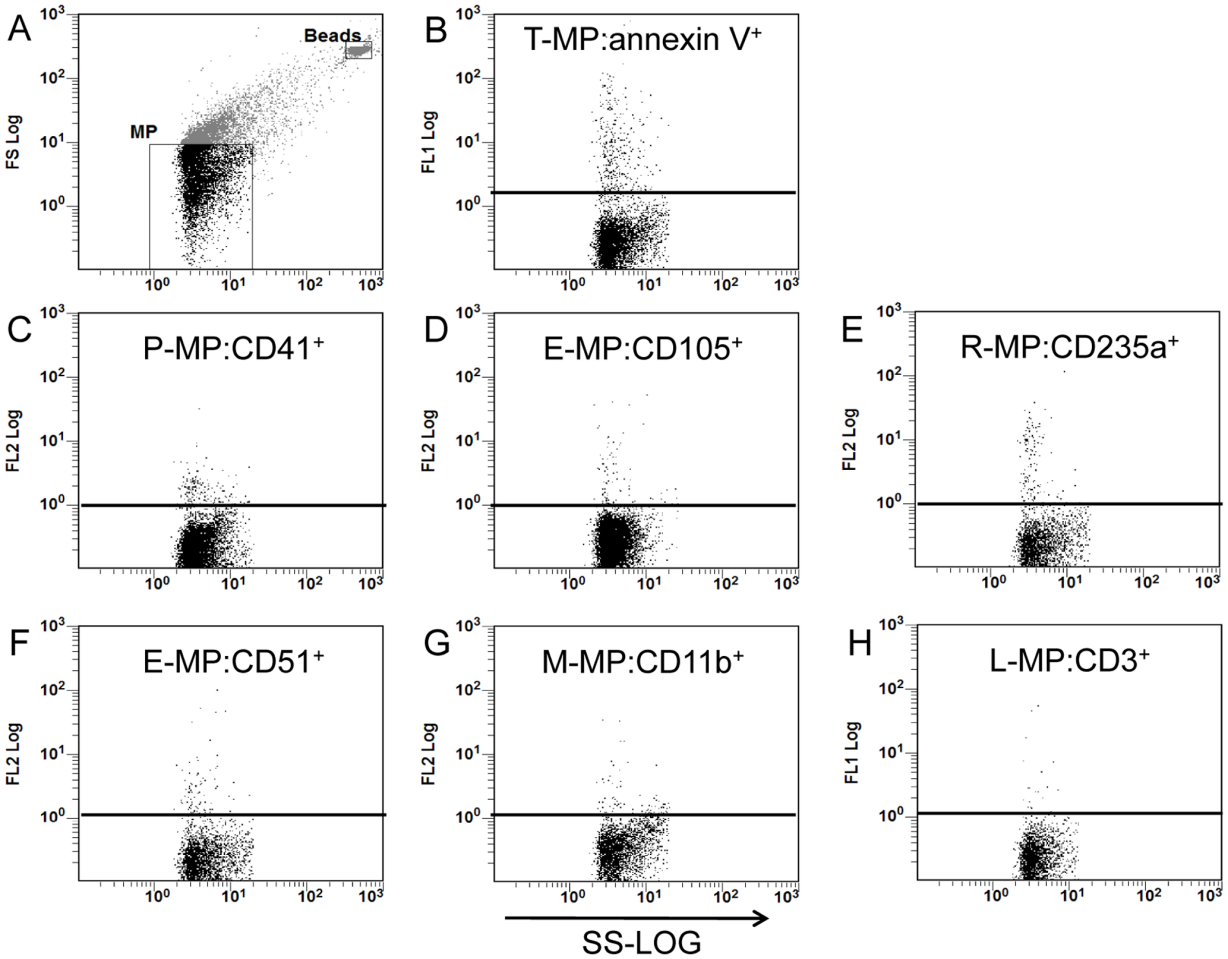
Flow cytometry analysis details and representative cytograms in a CM patient. MP were discriminated by their size and structures (Fig. **1A**). And events in MP gate were further analyzed to differentiate Annexin V^+^ MP from the background signal. An internal standard, constituted of fluorosphere beads of a known size and at a known concentration was used to help set the MP gate and calculate the MP level. Representative cytograms: **1B** to **1H** Illustrations of the differences in MP positive events in cytograms representing total MP and cell-derived MP in a CM patient. (**B**): T-MP:annexin V^+^; (**C**): P-MP:CD41^+^; (**D**): E-MP:CD105^+^; (**E**): R-MP:CD235a^+^; (**F**): E-MP:CD51^+^; (**G**): M-MP:CD11b^+^; (**H**): L-MP:CD3^+^.These MP positive events were used in the calculations of the number of MP/µl of plasma (see the methods section).

Labeled plasma samples were analyzed on a Beckman-Coulter FC500-MPL flow cytometer. The MP region was defined using a FSC-SSC dot plot (Fig. 1A) as described [12]. Data were acquired for 90 sec and positive MP were discriminated for their binding to specific mAb and annexin V. Representative cytograms of each type of MP, total and cell-derived, are shown in Fig. 1B–H. Flowcount® beads were distinct from the MP population and could thus be counted on the same cytogram (Fig. 1A). Absolute numbers of MP were then calculated by the following formula:
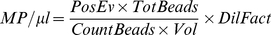
With :

PosEv = Number of positive events in LOG-FL/LOG-SSC; TotBeads = Total number of beads per well (20000); CountBeads = Number of beads counted in 90 sec; Vol = Test volume (240 µl); DilFact = Dilution factor (120).

### Statistical analysis

Data were analyzed using GraphPad-Prism v.5.00, GraphPad Software, San Diego, CA. A non-parametric analysis of variance (ANOVA, Kruskall-Wallis with Dunn's post-test) was used to compare several groups, while comparison of mean total and cell-specific MP levels between two different groups (paired samples) was analyzed with Wilcoxon test. Spearman's rank correlation coefficients were calculated to investigate the relationship between MP counts, and clinical as well as biological parameters. P<0.05 (*) was considered significant.

## Results

### Total and cell-specific MP levels increased on admission in CM patients

**Figure 2:**
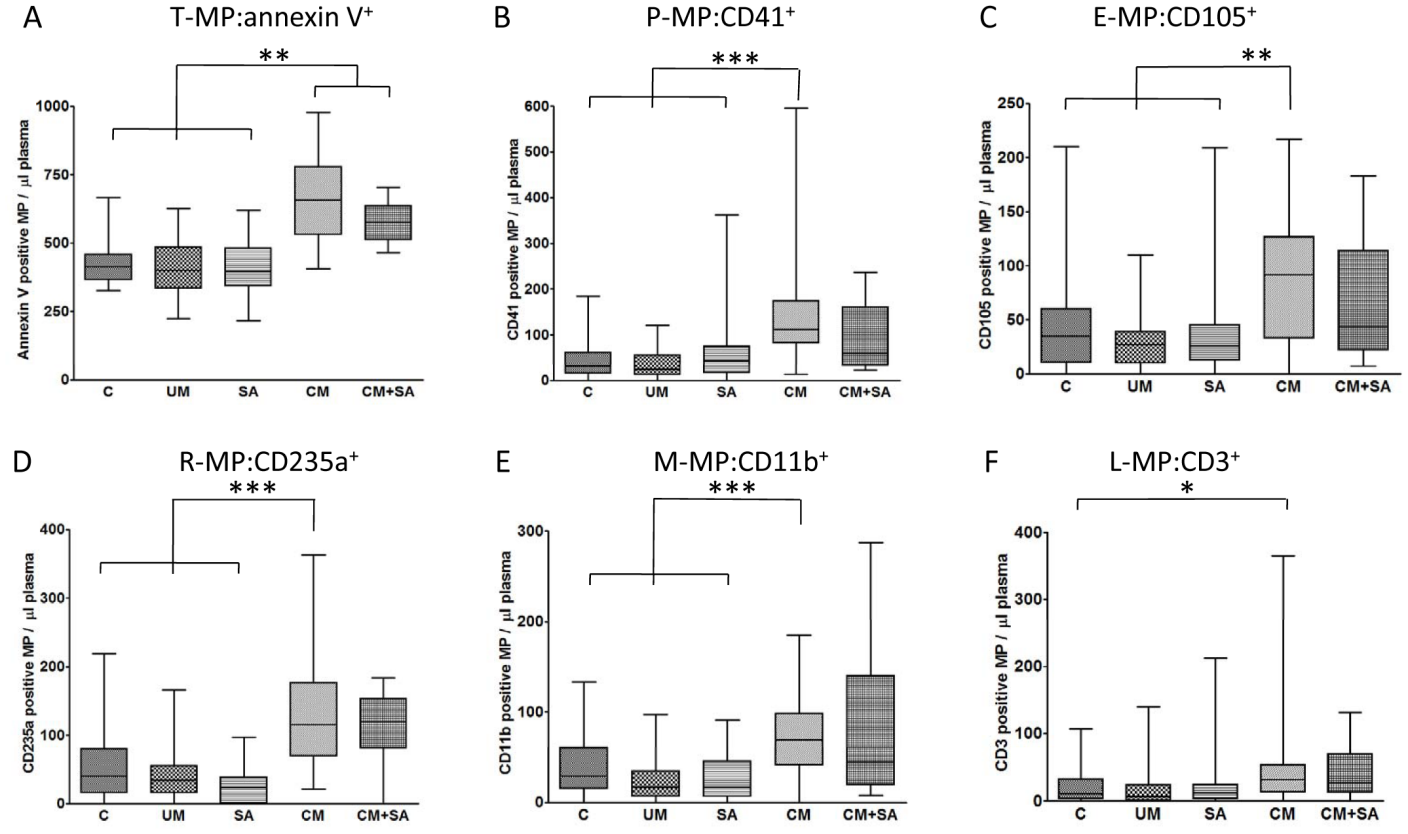
Total and cell-specific MP levels per µl of plasma on admission in the different groups. (**A**): T-MP:annexin V^+^; (**B**): P-MP:CD41^+^; (**C**): E-MP:CD105^+^; (**D**): R-MP:CD235a^+^; (**E**): M-MP:CD11b^+^; (**F**): L-MP:CD3^+^. Dot plots representations showing elevations of total MP and the different cell-specific MP investigated in CM and CM+SA patients. C: Controls; UM: uncomplicated malaria; SA: severe malaria anemia patients on admission; CM: cerebral malaria patients on admission; CM+SA: patients with the combine symptoms of CM and SA on admission; MP levels are given in MP/µl of plasma. *: p<0.05, **: p<0.001, ***: p<0.0001.

T-MP:annexin V^+^ levels were significantly increased in the groups with cerebral involvement (CM and CM+SA), when compared to controls, UM, and SA groups (Fig. 2A, 436±16, 405±13, 408±13, 656±28, and 580±24 MP/µl for control, UM, SA, CM and CM+SA respectively; P<0.001). The same trend was seen for all cell-specific MP studied with significant differences between the CM group and control groups (control, UM and SA) observed: P<0.05 for L-MP:CD3^+^ only between C and CM, P<0.001 for E-MP:CD105^+^, P<0.0001 for P-MP:CD41^+^, R-MP:CD235a^+^ and M-MP:CD11b^+^ subpopulations; Fig. 2B to F, respectively).

While each type of MP appeared to be represented equally within the control and UM groups, P-MP:CD41^+^ and R-MP:CD235a^+^ were the most prevalent in the CM and CM+SA groups, while P-MP:CD41^+^ were the most abundant subpopulation (not shown). In all groups, the L-MP:CD3^+^ were the least represented.

When analyzing for each group the percentage of patients that display elevated numbers of MP, i.e., above a defined threshold = mean of control group or mean of control group+2 SD (not shown), we found that CM and CM+SA were the only groups where at least 65 and 50% of the values, respectively, were higher than the control group mean, and between ∼10 and ∼50% were higher than mean+2 SD. It is worth noticing that in these 2 groups, the P-MP with levels superior than mean+2SD were found in 43.7 and 30% of the patients and 50 and 40% have elevated T-MP:annexin V^+^ levels. Endothelial cells release phenotypically and quantitatively distinct MP. Two distinct specific endothelial antigens were investigated. While E-MP:CD51^+^ numbers were lower than E-MP:CD105^+^ ones, a significant positive correlation was obtained between the two determinations (r^2^ = 0.4786, P<0.0001, not shown).

### Total and cell-specific MP levels at follow-up/discharge

To evaluate the relationship between MP levels and clinical improvement, another blood sample was collected from children with SA and/or CM, at discharge.

**Figure 3:**
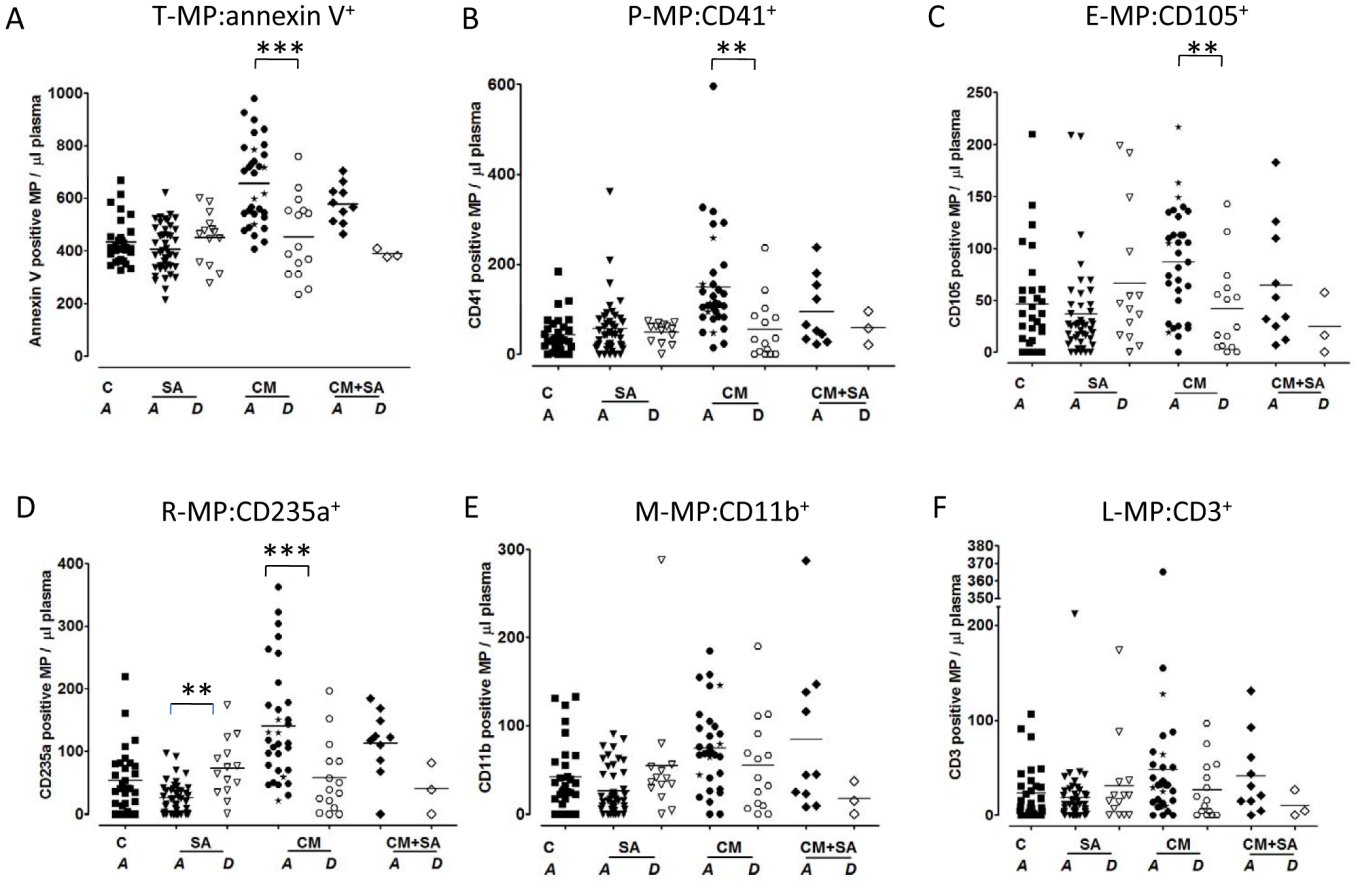
Total and cellular MP levels per µl of plasma at follow-up/discharge (7 days after admission: *A*) versus levels on admission. (**A**): T-MP:annexin V^+^; (**B**): P-MP:CD41^+^; (**C**): E-MP:CD105^+^; (**D**): R-MP:CD235a^+^; (**E**): M-MP:CD11b^+^; (**F**): L-MP:CD3^+^. Dot plot representations showing how in most of the cases, MP levels decreases in patients with CM at discharge (*D*). Control group is represented to show that patient levels are returning to control levels. C: Controls; SA: severe malaria anemia patients on admission; CM: cerebral malaria patients on admission; CM+SA: patients with the combine symptoms of CM and SMA on admission; SA(D): severe malaria anemia patients at discharge; CM(D): cerebral malaria patients at discharge; CM+SA(D): patients with the combine symptoms of CM and SMA at discharge; MP levels are given in MP/µl of plasma. The values of the deceased patients are presented as black stars on Figure 3. *: p<0.05, **: p<0.001, ***: p<0.0001.

In the CM groups, the high levels of T-MP:annexin V^+^ seen on admission dramatically decreased from 656±28 MP/µl on admission to 455±40 MP/µl (p<0.0009) at discharge to return within the normal range (Fig. 3A). When, only paired samples were analyzed, 13 out 15 (87%) were significantly lower on discharge (p = 0.0002). P-MP:CD41^+^, E-MP:CD105^+^ and R-MP:CD235a^+^ levels also all decreased significantly at discharge (Fig. 3B: p = 0.0004, C: p = 0.0099 and D: p = 0.0028, respectively). Again, when only paired samples were observed 13 to 14 out of 15 samples (87 to 93%) were significantly decreased (P-MP:CD41^+^: p = 0.0067, E-MP:CD105^+^: p = 0.007 and R-MP:CD235a^+^: p = 0.0005, respectively). Even though 53 and 60% of L-MP:CD3^+^ and M-MP:CD11b^+^ levels, respectively, were decreased at discharge, no statistical difference was observed (Fig. 3E and F).

In the SA groups, we found a significant increase in the R-MP:CD235a^+^ levels between paired samples at admission and discharge (25±4 MP/µl to 73±12 MP/µl, p<0.0035, Fig. 3D) where 11 out of the 14 samples (78%) displayed this increase. Although no statistical difference was observed total, L-MP:CD3^+^, M-MP:CD11b^+^ and E-MP:CD105^+^ levels also tended to increase at discharge.

Due to a low number of discharge samples, no significant difference was observed between the CM+SA groups, however it is also worth noticing that all MP levels decreased at discharge (Fig. 3A to F).

### Correlations between MP levels and clinical or biological parameters

**Figure 4:**
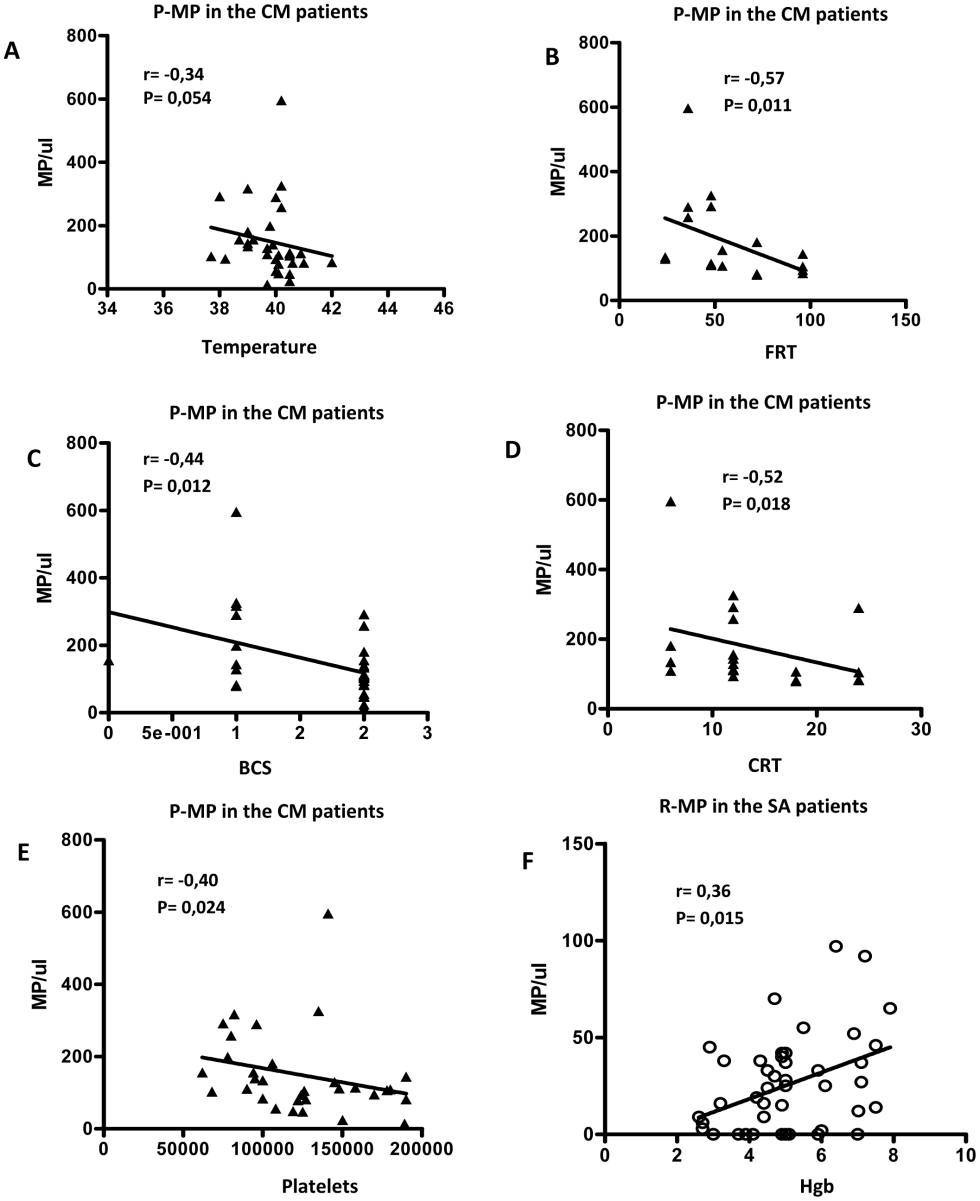
Correlations between MP and some clinical/biological parameters. A to E: correlations between P-MP numbers and temperature, fever resolution time (FRT), Blantyre coma score (BCS), coma resolution time (CRT) and platelet counts, respectively. F: correlation between R-MP and hemoglobin levels (Hgb).

We investigated possible correlations between MP and some clinical and biological parameters obtained during patient recruitment, namely age, temperature on admission, fever resolution time (FRT), Blantyre coma score (BCS), coma resolution time (CRT), prostration resolution time (PRT), parasitaemia, hemoglobin level and white blood cell, red blood cell, and platelet counts. In the CM group, we found negative correlations between P-MP:CD41^+^ and temperature (r = −0.343, P = 0.0545), FRT (r = −0.571, P = 0.0106), BCS (r = −0.437, P = 0.0122), CRT (r = −0.524, P = 0.0176), and platelet count (r = −0.397, P = 0.0244) (Fig. 4 A–E). In the SA group, hemoglobin level and R-MP:CD235a^+^ were positively correlated (r = 0.359, p = 0.0154) (Fig. 4F); however, there was no correlation between red blood cell counts and R-MP:CD235a^+^ levels in the SA group (r = 0.100, P = 0.5118) nor between parasitaemia and MP levels.

Due to the different numbers of males and females in some groups, we investigated total and cell-specific MP levels in males versus females. No statistical difference was obtained in any of the MP subpopulations.

### MP in CM fatal cases and in CM survivors

During the course of patient recruitment, death occurred in 5 patients, all in the CM group. We compared total and cell-specific MP levels in survivors and fatal cases. No statistical difference was obtained except for E-MP:CD105+, where we found 78.85±8.37 EMP/µl in those who survived compared to 130.6±33.14 E-MP:CD105^+^/µl in those who died (p = 0.0370). The values from the deceased patients are presented as black stars on Figure 3.

## Discussion

We report here, in patients with severe malaria, elevated MP numbers and, for the first time, a detailed analysis of their cellular origin. During the last ten years, a growing number of studies have described elevated numbers of MP subpopulations in association with various disease states, as well as studies investigating the composition and functional characteristics of MP [15],[12],[16]. Moreover, a role for MP in the pathogenesis of the disease is supported by the fact that, in experimental CM, (i) MP levels are increased in the plasma of mice at the time of the development of the neurological syndrome [7], (ii) the absence of MP overproduction is associated with protection against experimental CM without altering the parasitaemia [7],[17]. Knock-out mice deficient for the *abca1* gene that do not up-regulate MP production in response to vesiculation agonists, are fully protected against experimental CM and display normal levels of plasma MP when their wild type CM-susceptible littermates show the onset of the syndrome [7]. In addition, when treated with pantethine, a physiological substance and co-enzyme A precursor in the Krebs cycle, used in patients for its hypolipemic properties [18],[19],[20], infected mice are protected against experimental CM and do not show elevated levels of plasma MP as their non treated littermates do [17]. We had previously reported dramatically increased endothelial MP levels in the peripheral blood of Malawian children with malaria, and this increase was restricted to patients with CM [12]. Here we sought to better understand this finding by investigating, in another malaria endemic country, endothelial MP, but also total MP along with MP from lymphocytes, monocytes, platelets and erythrocytes.

In this study we have analysed separately the different types of MP. Single stainings have been performed for each marker (annexin V or monoclonal antibody). Several studies have described the presence of MP that do not bind annexin V [3], [21]. It is not known whether this is due to a lack of phosphatidylserine at the surface of MP or levels of phosphatidylserine that are undetectable by current flow cytometers. Since the significance of these annexin V «negative» MP remains to be elucidated, we have chosen to perform single stainings. Also, the antigens we have chosen to detect at the surface of MP are specific for cell types and there is no co-expression between the different types of MP analysed here. Although we have not performed double staining, since the number of annexin V positive MP for a patient is generally equal or superior to the sum of all MP detected with specific antibodies, it is likely that most of these MP are annexin V positive.

Total and cell-specific MP levels were found to be elevated in CM patients whereas only total MP levels were higher than controls in both groups with cerebral involvement (CM and CM+SA). Those increased levels suggest the activation of a broad range of immune cells during CM as well as their alteration, secondary to the cerebral processes [22], [23]. We found that M-MP:CD11b^+^, P-MP:CD41^+^, E-MP:CD105^+^ and R-MP:CD235a^+^ were increased in CM- associated vesiculation processes and, to a lower extent, L-MP:CD3^+^. In the murine model of CM, brain vessels at the time of the neurological syndrome showed focal accumulation of monocytes [24] and platelets. The latter are seen in both human and murine CM [25],[11]. Pf-infected erythrocyte sequestration in the microvasculature is the major feature of CM histopathology leading to the disruption of the blood-brain barrier. This sequestration is observed in almost all organs, including the brain [26] and could explain increased MP levels which could be specific to malaria with cerebral involvement. In this particular study, determination of the parasite load via assaying the parasite-derived lactate dehydrogenase (pLDH) in the post-mortem tissues [27] was not performed as the ethical consent in Cameroon was only for blood samples and the number of deceased patients was low (n = 5). In the Malawian study [27], the results of the p-LDH assay had suggested that levels of sequestration are higher in most organs for patients who died of CM than patients with assumed CM then diagnosed as non CM at autopsy.

MP carry at their surface proteins able to allow their binding to target cells, but also mediate cell–cell interactions through cell adhesion molecules or simple contact. Moreover, MP are able to transfer antigens from their originating cell to another [3],[28],[29]. Thus, the cells involved in the vesiculation increase are also those involved in sequestration [5].

In SA patients, we observed a decrease in R-MP:CD235a^+^. SA is due to the destruction of both parasitized and non-infected erythrocytes [30]. A greater number of R-MP:CD235a^+^ in SA patients could be expected, as for the thrombocytopenia seen in CM, but was not observed. This could be explained by a long time-course pathology with chronic and/or repeated infections that has lead to a gradual decrease of RBC and hemoglobin concentration [31],[32]. This may also result in the clearance of MP, and SA patients might have consulted after their R-MP had already been cleared by the immune system [33].

Total and cell-specific MP were also investigated when children were discharged. In children with CM, MP were decreasing and, for most of them, returned to control values. Due to small patient numbers, statistical tests did not reach significance for the CM+SA group even though a marked decrease was observed between admission and follow-up values in the whole population and in paired samples. This is consistent with previous findings where E-MP:CD51^+^ levels returned to normal at convalescence [12]. Apart from immune MP clearance, these MP level reductions in discharged CM patients could be associated with the disappearance of neurological manifestations such as coma, fits or palsies [2].

By contrast, in the SA group, we observed a trend to increased MP levels at discharge. SA patients with low RBC count and hemoglobin concentration on admission received whole blood transfusions before and between their anti-malarial therapies. Blood packs were obtained from donors at the blood bank of hospitals. Aside from cold storage, blood packs were not submitted to any processing. MP shedding is known to increase spontaneously at 4°C and stored platelets shed more MP than fresh platelets do [34]. We can thus hypothesize that the observed increase of cell-specific MP levels in SA patients could be due to the MP generated during the storage of blood prior to transfusion, and/or some anaphylactic reactions due to blood incompatibilities in the recipient incidental to the transfusion [35].

Circulating MP levels were found to correlate with a number of clinical and biological parameters. In CM patients, the P-MP:CD41^+^ increase was associated with the depth and duration of the coma, as indicated by the negative correlations between P-MP:CD41^+^ levels and BCS and CRT. In our clinical setting, one could not predict the coma duration based solely on how deep the coma was. Indeed, the cause of impaired consciousness was generally unclear and coma likely to result from several interacting mechanisms [2]. In addition, a negative correlation between P-MP:CD41^+^ and platelet counts was found. This suggests that CM-associated thrombocytopenia is characterized by P-MP release. This is consistent with previous studies in the murine model and supports the view that platelets are involved in the neurovascular injury during CM [25],[11]. It is tempting to hypothesize that, despite a marked thrombocytopenia, attributed to both platelet destruction and platelet sequestration in various organs [11],[36], these P-MP:CD41^+^, because they are still present in the circulation, could be responsible for some of the platelet-related effects such as activation of coagulation or adhesion. All these processes would contribute to the neurovascular lesions and emphasize the pivotal role of platelets in CM pathogenesis [3].

While the microvascular sequestration primarily involves infected erythrocytes, there was no correlation between parasitaemia and R-MP:CD235a^+^ levels. It is noteworthy that in malaria endemic contexts, most patients coming to hospital have already taken some treatments (self medication prior to admission), a factor which may influence the parasitaemia assessment [37]. Also, no link was observed between MP levels and gender supporting the idea that vesiculation changes occur indiscriminately in both genders, as CM symptoms and signs do. In conclusion, we report here the presence of higher levels of total and cell-specific MP in CM patients but not in those with SA or UM and clearly demonstrate their value as a biomarker in the CM pathogenesis. To our knowledge, it is the first time that the diverse cellular origins of MP and their association with some cerebral dysfunctions and biological parameters have been investigated in pediatric severe malaria. This study validates that sequestration occurring in deep microvessels during CM involves a broad range of vascular cells, particularly platelets, erythrocytes, monocytes and endothelial cells. MP, and most notably those from platelet origin appear to be a relevant marker in the follow up of patients with CM because (i) their levels were the highest, (ii) they correlated with important clinical and biological parameters such as coma score and platelets counts and (iii) they returned to normal values when the patient was cured, making this parameter a potentially useful indicator of the efficiency of patient management. MP enumeration is a simple and rapid assay that could be implemented in hospitals dealing with malaria patients and equipped with a flow cytometer to allow analysis on fresh plasma or even whole blood. Further kinetics studies are required to define whether elevated MP numbers can be considered as a predictive marker of severity for malaria patients.

Altogether, the previous results on the pathogenesis of MP in experimental CM and the patient data generated in this study warrant the use of MP as biomarker during severe malaria infection and support further pharmacological studies aiming at decreasing their production.
